# “*I can’t fail. And my son can’t fail.”:* Caregiver perspectives on supporting youth to self-manage their HIV in Ndola, Zambia

**DOI:** 10.1371/journal.pgph.0005416

**Published:** 2025-11-20

**Authors:** Kirsty M. Sievwright, Haneefa Saleem, Kayayi Chibesa, Kristin Mmari, Christiana Frimpong, Sam Miti, Jonathan K. Mwansa, Julie A. Denison

**Affiliations:** 1 Department of International Health, Johns Hopkins Bloomberg School of Public Health, Baltimore, Maryland, United States of America; 2 HIV Center for Clinical and Behavioral Studies, New York State Psychiatric Institute and Columbia University, New York, New York, United States of America; 3 University of Lusaka, Lusaka, Zambia; 4 Arthur Davison Children’s Hospital, Ndola, Zambia; 5 Department of Population, Family, and Reproductive Health, Johns Hopkins Bloomberg School of Public Health, Baltimore, Maryland, United States of America; PLOS: Public Library of Science, UNITED STATES OF AMERICA

## Abstract

Adolescents and young adults (AYA, aged 15–24) living with HIV have suboptimal antiretroviral treatment adherence compared to their adult counterparts, raising concerns of poor health and drug resistance. While it is known that AYA require caregiver and family support to successfully transition to HIV self-management, there has been limited engagement with caregivers to this end. To fill this gap, we examined caregivers’ experiences of navigating their youth’s HIV self-management to better inform interventions that seek to improve AYA’s HIV self-care and treatment outcomes. We conducted a thematic analysis using qualitative in-depth interview transcripts from 21 caregivers of AYA living with HIV who participated in the *Project Yes!* study, which was a randomized control trial to test a clinic-based youth peer mentoring program in Ndola, Zambia. We used inductive and deductive approaches to explore caregiver experiences, roles, and needs related to their youths’ HIV self-management. We found that caregivers’ commitment and willingness to support their AYA shaped how they considered and prioritized their AYA’s health and well-being. Further, caregivers shared their views on approaches to communicating with their AYA, namely the benefit of communication that is gentle, encouraging, and grounded in knowledge of their youth. Finally, caregivers affirmed their desire for their AYA to successfully *“take care of [themselves]”* and described how to effectively support their AYA to start assuming primary responsibility by giving their youth space to self-manage with caregiver oversight. Our analysis affirms the valuable role caregivers play in youth’s HIV self-management. Based on these findings, we offer important insights to encourage and inform future research and interventions that support and engage caregivers to facilitate their AYA’s transition to HIV self-management.

## Introduction

Adolescents and young adults (AYA, aged 15–24) living with HIV often experience more incomplete antiretroviral treatment (ART) adherence compared to their adult counterparts [1–4]. This reality is partly due to young people’s developmental stage and ongoing cultivation of their understanding, skills, and confidence, as they take on more responsibilities to self-manage their HIV care and treatment [2–4]. HIV self-management is the combination of processes and behaviors necessary to successfully manage a chronic condition such as HIV (e.g., determining care needs, engaging routinely in care, and modifying lifestyle) [5,6]. This also includes ART adherence to achieve HIV viral load suppression and to ensure that AYA have future care options available to them [2,4,7]. AYA, however, continue to have the lowest rates of viral load suppression in Zambia, an estimated 70% compared to 86% among all people 15 and older living with HIV [8]. This disparity in viral load suppression highlights the need to support AYA as they transition into adulthood and take on more HIV self-management responsibilities.

For AYA living with HIV to successfully transition to self-management, support is needed from their caregivers at the family and household level [6]. A lack of social support is associated with incomplete ART adherence among AYA, challenging their ability to take their daily treatment [3,4,9]. The family and household notably influence AYA’s HIV behaviors and outcomes, in part because in Eastern and Southern Africa they are often the only people who know the AYA’s HIV status and are uniquely positioned to provide support [9–15]. These findings correspond with the Pediatric Self-Management Framework, that illustrates how caregivers and other family and household members play an important role in AYA’s chronic illness health and care management [5] with caregivers often ensuring their AYA has the instrumental, informational, appraisal, and emotional support to successfully live with HIV [10,16]. Moreover, caregivers are positioned to play a central role in their AYA’s transition to HIV self-management by shaping their AYA’s behaviors, knowledge, and self-efficacy to self-manage as well as handing over these responsibilities to the youth [17–20]. However, caregivers may not always feel capable of supporting this transition. In Zambia and Uganda, caregivers reported experiencing uncertainty and difficulties with when and how to let their AYA take more responsibility for their own HIV care and management [6,10]. In research studies in Zambia, the caregivers of youth living with HIV were mainly described as mothers aged in their mid-40’s; however, a range of characteristics and biological and non-biological relations were represented [21].

There is a noted gap in the literature on the engagement of caregivers in the context of AYA HIV self-management in Eastern and Southern Africa [6,10,15,22,23]. Most programs to date, especially among AYA ages 15 and older, center solely on the AYA [7,24]. Most caregivers are navigating their role without programmatic support and, often in isolation, without friends or family who are aware that their youth is living with HIV [10]. This analysis addresses this gap by understanding the experiences and views of caregivers with AYA living with HIV on how to manage the transition to self-management and support their youth.

## Methods

### The *Project YES!* Youth Engaging for Success study

Our analysis utilized in-depth interview data from adult caregivers of AYA who participated in the *Project YES!* study [25,26]. *Project YES!* was an individually randomized controlled trial (RCT, NCT04115813) that took place between December 15, 2017 and June 1, 2019 in Ndola, Zambia. *Project YES!* aimed to support AYA living with HIV to achieve viral load suppression and reduce internalized HIV-related stigma through a clinic-based youth peer mentoring program with a caregiver component. This Centers for Disease Control and Prevention evidence-based intervention and RCT has been described in detail elsewhere [25,26]. Regarding caregiver involvement in *Project YES!*, AYA in the intervention arm were able to invite an adult caregiver to attend an orientation meeting and to participate in up to three voluntary caregiver group meetings. These meetings focused on improving caregiver’s capacity to support AYA living with HIV as they transition to HIV self-management, through information sharing and group discussions on: the basics of HIV (definitions, AYA’s sexual and reproductive health), HIV stigma and discrimination (definitions, mitigation strategies, disclosure), and flourishing with HIV (HIV treatment and adherence, nutrition, and community resources) [25–27].

The purpose of the in-depth interviews with caregivers in the parent study was to examine caregivers’ experience with the *Project Yes!* intervention and the mechanisms related to AYA achieving viral load suppression. Adult caregivers were eligible to participate if their purposively sampled AYA participated in an in-depth interview (n = 41) and gave permission for the study team to invite the caregiver to join. In-depth interviews with 23 caregivers were conducted by three trained, Zambian interviewers in English or Bemba using a semi-structured interview guide (S1 Text: Interview Guide). Topics covered in the interview guide included the caregiver-AYA relationship, AYA HIV self-management, the caregiver’s HIV self-management support role and needs, and experiences with *Project YES!* caregiver support groups. The interviewers for the *Project YES!* study were hired based on their experience working with young people; part of their training included rapport building with cargiver participants. The interviewers obtained written informed consent from the respondents prior to the interviews. The in-depth interviews took place at one of the four participating health facilities and lasted on average 40 minutes. Participants were reimbursed 50 Zambian Kwacha (approximately 5 USD). Interviews were audio-recorded, transcribed, and, as needed, translated into English.

### Data analysis

We used transcripts from 21 caregivers in this inductive and deductive thematic analysis [28]; we restricted the sample to include only caregivers who were older than and not married to the participating youth. Two members of the study team (KMS and CF) inductively double-coded two transcripts and developed an initial inductive codebook. This step was followed by an iterative process in which the lead author (KMS) inductively generated and applied codes to all the remaining transcripts using Atlas.Ti 8.4. The resulting codebook was then refined to include deductive codes based on the Pediatric Self-Management Framework by Modi et al. (2012) and key domains in the semi-structured interview guide [5]. The lead author then applied the deductive codebook to all transcripts in a second round of coding. She iteratively memoed and utilized matrices and thematic maps to identify, define, and reevaluate emergent themes and subthemes. She also made group-level comparisons between caregivers of late adolescents (aged 15–19 years) and young adults (aged 20–24 years) as well as between caregivers who identified themselves as the AYA’s mother and as another relation to the AYA. The lead author discussed the analytical processes, emergent findings, and her positionality with co-authors throughout analysis to incorporate their metholodological and contextual expertise as well as ensure the credibility and confirmability of this sub-analysis. She also shared and discussed the findings with the peer mentors and the staff at one of the hospitals that participated in the *Project YES!* study to further ensure the confirmability of the results.

### Ethics statement

The *Project YES!* study was approved by the Zambian Ministry of Health through the National Health Research Authority as well as the ethical review boards of ERES Converge in Zambia and the Johns Hopkins Bloomberg School of Public Health in the United States.

### Inclusivity in global research

Additional information regarding the ethical, cultural, and scientific considerations specific to inclusivity in global research is included in the Supporting Information (S1 Checklist).

## Results

The characteristics of the study sample are described in Table 1. Most of the respondents were female (n = 18) and identified as their AYA’s parent (n = 12). The majority had female (n = 13) and 15–19-year-old (n = 16) AYAs. About half of the respondents attended two or three of the four *Project YES!* caregiver meetings (n = 11); one caregiver attended the orientation and all three meetings; and five attended none. While caregivers were not all asked about their own serostatus, eight caregivers disclosed that they were also living with HIV.

**Table 1:** Respondent characteristics stratified by relationship to youth (N = 21).

	Total (N = 21)	Caregivers who identified as the AYA’s mother	Caregivers who identified as a relation other than the AYA’s mother
**Sex**			
Male	3	0	3
Female	18	11	7
**Age (years)**			
Mean (Range)	45.3 (23-64)	45.73 (36-62)	44.9 (23-64)
25 and younger	1	0	1
26-35	2	0	2
36-45	8	7	1
46-55	5	2	3
56-65	5	2	3
Relationship to AYA*			
Parent	12	11	1
Grandparent	3	0	3
Step-Parent	1	0	1
Aunt/Uncle	3	0	3
Sibling	1	0	1
Cousin	1	0	1
Total number of CG meetings attended**			
0	5	1	4
1	4	2	2
2	4	3	1
3	7	5	2
4	1	0	1
**Sex of AYA**			
Male	8	6	2
Female	13	5	8
**Age of the AYA**			
15-19	16	8	8
20-24	5	3	2
**AYA self-reported mode of HIV acquisition**			
Perinatal	17	9	8
Sexual	1	1	0
Other	1	0	1
Don’t Know/Missing Response	2	1	1

* This reflects how the caregiver identified their relationsip to the AYA , not necessarily how they are biologically related.

**Total number of meetings includes any combination of the orientation and 3 subsequent meetings, ranging from 0 to 4.

Through this analysis, three key themes emerged regarding caregivers’ experiences with AYA transitioning to HIV self-management: (1) commitment to meet the needs of AYA living with HIV, (2) considerations for communicating with their AYA living with HIV, and (3) building capacity and confidence in AYA self-management.

### *“[W]here health is concerned, we can come”:* Caregivers’ commitment to meet the needs of AYA living with HIV

Nearly all respondents conveyed a commitment to their youth’s health and well-being as well as a willingness to meet their youth’s needs, particularly among those caregivers who attended *Project YES!* sessions and were the AYA’s mother. Several caregivers shared how their desire to support their AYA’s health and well-being motivated their actions and decisions. This sentiment was most salient in discussions on why caregivers chose to participate in *Project YES!*, with many to wanting to improve their knowledge and capacity to more effectively care for their youth.

*“What was encouraging us is that we knew that what we were coming to learn here would help us take care of these children and we need to do those things at home.”* – Mother, 36 years old

*“[Regarding attending Project YES!,] where health is concerned, we can come. Because we want to know how we can keep our children, our nieces. […] Because we are talking about health.”* – Uncle, 52 years old

Other caregivers attended *Project Yes!* to show their youth they cared about them. This sentiment was expressed by one mother (aged 52 years old): *“So I wanted to support her [...] For her to see that she is not alone. I didn’t want to miss.”* As well as by an Aunt (aged 38 years old), who shared that she was motivated to *“immediately come”* to the *Project YES!* meetings because she knew her niece wanted her to join; she did not want her niece to *“start feeling bad”* if her aunt did not attend nor think about how she does not have a mother. Many of these discussions reflected an awareness of the impact caregivers can have on their youth living with HIV and of the responsibility that their role as caregivers presents.

Commitment to their youth’s health and well-being was also conveyed by several caregivers in the context of financial considerations and obstacles. Several caregivers noted that even if they were not reimbursed for transportation to *Project YES!*, they would still have found a way for them or their AYA to attend. These caregivers valued opportunities that benefited the health and well-being of their AYA living with HIV, such as what was offered through *Project YES!*. Three of these caregivers were particularly resolute that they would find the means necessary to meet their youth’s health needs, whether to attend a program like *Project YES!* or to pick up medication. As one Mother (aged 36 years) noted: *“Even if things are [financially] difficult, I can’t fail. And my son can’t fail.”*

### *“Children need to be encouraged”*: Caregiver considerations for communicating with AYA living with HIV

Most caregivers shared their views or experiences regarding communicating with their AYA living with HIV, stressing *“gentle”* and supportive communication, and avoiding *“harsh”* and discouraging interactions. Being gentle was often discussed as beneficial for facilitating their youth’s health and well-being in the context of HIV (e.g., encouraging adherence or to attend clinic appointments), while being harsh was described as counterproductive to this end.

*“[Parents] should not be bitter. They should not get tired; they should just have the attitude of encouraging that child […] Because for children, if they are not encouraged, they will not take their medication.”* – Step-Mother, 54 years old

*“If the child is not finding it easy to come for review or to collect medicine, that means you don’t support. Maybe you shout at the child, like ‘Go for review [at the clinic], that’s why you are sick.’ Such things. Because they will be thinking, ‘That’s what mum is saying, I will not go for review.’ If you support the child and you talk to them in calm voice, the child will know that my mum supports me [and the child will go for review].”* – Mother, 52 years old

Several respondents also noted the importance of offering reassurance to their youth, including reminders that they are *“not the only one”* living with HIV and that they can accomplish the same things as a person who is not living with HIV. Encouragement often pertained to the importance of adherence. Some caregivers would speak frankly about the consequences of not taking medication on time and consistently, and remind their youth about the benefits of maintaining adherence.

*“[I]f you are missing some days [of your medication], that means you are putting your life at risk. You can die, you can’t live longer. Your father and I are also on medication. [...] That’s why we are still alive. We want you also to live longer like us.”* – Mother, 36 years old

*“Sometimes there are things like… like my son, sometimes he asks if it’s possible for him to have his own family in his status. […] I try to explain to him that [… y]es, you can have your own family. As long as you take your medication just as you are told, without missing. […] If you stop taking your medication, then you are ruining your life.”* – Father, 47 years old

Some caregivers also emphasized the value of *“knowing how to deal with”* and understanding of their AYA. Such knowledge and understanding were described as invaluable in gauging the youth’s mood or needs as well as to shape interactions that would facilitate a productive discussion.

*“Those who discuss with the children should not be harsh with them [... T]hey need to get to know the child, that today he is in a bad mood for them to start discussing with him. […] So as parents, if you also make them upset, you will not solve anything. So you need to observe that today he is in a bad mood, so you will start to be gentle with them. […] And when they normalize, then you can tell them what they need to be told.”* – Mother, 36 years old

*“You don’t need to shout at them. You need to know how to deal with them, because if you keep shouting at the child they may even stop taking medication.”* – Aunt, 38 years old

A few caregivers shared that when they communicated well and *“[got] along with”* their youth, more successful engagements would follow, allowing them to better respond to their youth’s needs.

*“We get along just fine. If she has a problem, she comes to ask, Mummy what should I do here? And then I tell her, this is what you are supposed to do. […] She is open because I am patient with her. So, if you treat a child harshly like shouting at them all the time, they can’t be free with you. So, what’s good with a child is that when you see something has happened, you call them and discuss nicely with them. You tell them, ‘Here you have made a mistake, you will need to do this and this’, or you tell them, ‘You did well on this and this and you need to continue doing this.’”* – Mother, 56 years old

### *“[What] she needs to really understand is how to take care of herself”:* Caregiver insights on building capacity and mutual confidence in AYA self-management

Nearly all the caregivers indicated that they had primary responsibility for or taken an active role in supporting their AYA’s HIV management. However, most respondents, regardless of the age of their youth, also stressed the importance of their AYA learning and managing to *“take care of themselves”*. This sentiment was often expressed as a desire for their youth to accept their HIV status or to be competent and effective in performing different HIV self-management behaviors (e.g., adhering to medication, attending clinical reviews, and safely navigating sexual relationships).

Several caregivers also shared how supporting and giving space to their AYA allowed greater ownership by the youth over their HIV self-management, while caregivers continued *“paying attention”*. Most of these caregivers, generally the AYA’s mother, paid attention to whether their youth followed through on the behavior in question (i.e., had taken their medication or attended their clinic appointment). Some would specifically do this by holding on to their AYA’s medication or appointment card and waiting for the AYA to come to them rather than proactively offering it. By being aware of, but not actively managing these aspects of their youth’s HIV care, some of these caregivers suggested they maintained opportunities to intervene (e.g., remind them to take their medication or attend their appointment) if the AYA failed to manage their HIV. However, most caregivers found that their youth was successful with attending appointments or taking medication.

*“I support him because I keep his medicine in our bedroom […] He takes in the morning and in the evening. So, what I want is that even if I’m sleeping or in the morning when he is leaving […] I like to see when he is taking his medication [...] I don’t remind him […] He just comes himself. He knocks at the door. He enters and takes his medication.”* – Mother, 36 years old

*“Because even when it comes to collect medicine, she does not delay. In the past, I would always have to remind her and to keep the date in mind for her. She would not even have the date in mind, that on this date I will go and collect. But now [since Project YES!], I have seen a difference. [...] As soon as the date comes, she will say, Mummy, you should give me the card I go and collect medicine. I realized this one is learning. Now she knows.”* – Mother, 36 years old

The importance of paying attention to their youth’s self-management practices is reinforced by one mother who prematurely thought her daughter was ready to manage her adherence. This caregiver’s experience, described below, suggests that paying attention to AYA as they start to assume greater ownership of their HIV care and management may serve as a valuable step in shifting the locus of responsibility and supporting youth’s self-management.

*“In the past we thought that she had grown a bit and she would take the medicine on her own. But then she was not taking. She would come and take when she wants. But now we have been encouraged through [Project YES...] At first, I didn’t know that she was not taking her medicine until I observed that we would come and the CD4 would not be good. Then I felt bad when we came to [the] ART [clinic] and they explained to me that the reason that she is having all these complaints is that she is not taking the medicine properly.”* – Mother, 42 years old

## Discussion

Our findings have important implications for future research and interventions to support caregivers’ ability to facilitate HIV self-management among AYA. This includes the significance of leveraging caregivers’ commitment and willingness to act to support their AYA’s health and well-being; building caregivers’ communication skills to facilitate constructive interactions between caregivers and their youth; and, finally, encouraging an engaged approach to support the gradual transition of care management from the caregiver to AYA.

The commitment and willingness demonstrated by caregivers of youth participating in *Project YES!* provides a foundation to facilitate AYA HIV self-management. These attributes can prompt caregivers to seek HIV knowledge and skills and to ensure that their AYA receives necessary healthcare and attends HIV-focused programs like *Project YES!*. Such actions on the part of caregivers enable AYA to develop the abilities and capacity to self-manage their HIV. While the AYA HIV literature has established the benefits of caregiver involvement more generally on the health and well-being of AYA living with HIV [10,29], our study takes it further to understand this commitment and willingness to take action from the caregiver’s perspective. Such insights have been limited in the HIV literature in the Eastern and Southern African context and are needed to advance research and interventions that engage the family and household level to support AYA living with HIV. In a reality in which healthcare systems globally are overstretched and unable to fully meet the needs of youth, and self-management behaviors most often occur in AYA homes, caregivers are invaluable resources. Engaging caregivers in programs like *Project YES!* is critical for fostering caregiver support, especially as the literature also shows that many caregivers struggle to transition HIV care management duties to their AYA [6,10,30,31]. For example, a previous study conducted in Zambia found that caregivers of AYA living with HIV “struggled in letting youth assume responsibility for their medication” [10], with some specifically noting that they did not want to “take chances” with their youth’s health [10]. We expect this to be particularly salient for caregivers of youth with perinatally acquired HIV, as these caregivers may have been involved with managing their youth’s HIV care for a significant portion of the youth’s life.

Our analysis also provides details from the caregivers’ perspectives on positive and constructive communication with AYA. It has been established that severe communication with AYA living with HIV can be counterproductive [6,15,32–35] and the values of parental warmth and positive communication have been maintained in the literatures on adolescent development, HIV prevention, and sexual and reproductive health [36–43]. However, in the available literature on AYA living with HIV in Eastern and Southern Africa, caregivers have typically expressed the need to be strict or penal for the sake of their youth’s health [6,32,33,44]. In our analysis, we observe a departure from this mindset as well as gain further insights from the perspective of the caregiver into how positive caregiver communication can play out with AYA living HIV in this setting. Respondents did not directly attribute their perspectives on caregiver communication to their engagement in the *Project YES!* intervention, nor was there dedicated sessions on communication in the caregiver group meetings; though it is possible that some caregivers views shifted through discourse with their fellow caregivers in these group meetings. These findings offer valuable insights to guide future interventions with caregivers in this context; as caregivers appear to have foundational understanding of the importance of positive and constructive communication with their youth, greater emphasis should be placed on supporting caregivers to further developing the skills necessary for effectively communicating with their youth.

Youth living with HIV are often not well transitioned into adult care and to self-management, which puts them at risk of adverse health outcomes [6,17,45–47]. These transitions to adulthood that take place within the clinic and family settings are generally “rushed” and the AYA is ill-prepared to successfully self-manage their HIV. For example, one study in Uganda observed that, from the perspective of some adolescent respondents, the transition to self-management took place in a “unplanned, abrupt manner” because caregivers were too busy to prepare the youth for this transition or to continue to manage their youth’s HIV care [17]. Within our analysis, caregiver respondents described a comparatively gradual approach to transitioning AYA to self-management within the family domain that would be beneficial to incorporate in to programs to support AYA living with HIV. This approach aligns with the idea of ‘scaffolding’ that allows youth to have opportunities to develop and hone skills (which in this case, would include those necessary for HIV self-management) with minimal interference from caregivers [36,48,49]. Scaffolding is a practical approach, fostering youth’s autonomy and behavioral self-efficacy while offering the safety net of their caregiver, who can monitor the AYA’s behaviors and intervene if necessary to prevent serious consequences. Further, the caregiver builds mutual confidence in their youth’s abilities such that they feel more comfortable shifting primary responsibility of their youth’s HIV care management over to them.

These findings have several important implications for the field. They reinforce the importance of *Project YES!* and future interventions that include caregivers of AYA living with HIV. Engaging caregivers to this end is inline with the Pediatric Self-Management Framework, which underscores the important role that caregivers and families play in supporting youth’s successful self-management [5]. We recommend that interventions engage such caregivers as partners in youths’ transition to self-management, providing opportunities and resources to amplify the benefits of their commitment and willingness to support their AYA. As noted by Heath et al. (2017), there is a need to support caregivers as they navigate this transition and the change in their caregiving role [50]. Specifically, we recommend including caregiver communication and scaffolding approaches in skills building, information-based, or counseling interventions that engage family and household members of AYA living with HIV. For example, group sessions with family members could be employed to discuss and model constructive ways to communicate with AYA living with HIV, including the gentle, encouraging, and knowledgeable forms of communication [51]. Further, scaffolding could be encouraged through a counseling approach that engages caregivers and family members along with their AYA, and facilitates discussions and planning around when and how to begin the AYA’s transition to self-management. Our results suggest that such programmatic efforts would benefit the caregiver and AYA alike in the process of transitioning to HIV self-management.

### Strengths and limitations

It is important to consider the findings of this analysis alongside several limitations. First, this was a sub-analysis of an RCT and the qualitative *Project YES!* data was collected for a different purpose than the one set forth in this analysis. This may have limited our ability to make group-level comparisons, as caregiver participants were not purposively sampled, but rather identified in relation to AYA qualitative interview participants. As our sample was predominantly comprised of caregivers of AYA with perinatally acquired HIV, we were not able to discern notable differences based on mode of acquisition. Further, no salient differences were identified between caregivers of late adolescents (aged 15–19 years) and young adults (aged 20–24 years) and only a couple of differences were discerned between caregivers who identified themselves as the AYA’s mother and as another relation to the AYA. We would advise that future studies deliberately sample on these characteristics to understand any key differences that may inform intervention development and implementation. Further, this research would have been strengthened by applying a Life Course Perspective to gain insights into how key transitions and developments for caregivers and youth (e.g., shifts in HIV care responsibilities, youth forming careers and romantic and sexual partnerships) have shaped caregivers’ support of and engagement in their youth’s self-management [52]. We recommend primary data collection applying this perspective in the future. Additionally, all the respondents were caregivers of AYA who had participated in *Project YES!* and, in most cases, had participated in the caregiver sessions themselves. As such, we must acknowledge a potential selection bias and that the caregivers in this analysis may differ from caregivers who were not involved in *Project YES!* or, more broadly, from those who are not as involved with their AYA in general. Further, given that these interviews were in the context of *Project YES!*, respondents, particularly those who were *Project YES!* participants themselves, may have been influenced to reiterate the lessons that they and their AYA were exposed to through *Project YES!* in their interview. The *Project YES!* study team sought to mitigate this response bias through stressing to respondents that they were interested in their individual feedback and insights to improve the program for the future.

This analysis does have several noted strengths, chief among them that it provides much needed data on caregiver perspectives and experiences with AYA HIV self-management, particularly in the context of an intervention. Caregiver affiliation with *Project YES!* was also a strength allowing us to highlight additional kinds of support caregivers and their youth may need, beyond that already offered through *Project YES!*, such as employing a scaffolding approach to self-management. Further, this study captured the perspectives and experiences of engaged caregivers, a consistently underutilized source of support for AYA transitioning to HIV self-management.

## Conclusions

Caregivers play an important role in AYA’s HIV care management. Recognizing the challenges that come with ‘letting go’ of their responsibilities, our study found that caregivers have a deep commitment for their youth’s health and are willing to follow through on actions needed to support their AYA living with HIV. Further, we highlighted caregivers views and approaches to constructively communicate with their youth living with HIV and begin to gradually transition the primary responsibility for HIV care management to the youth. Moreover, based on these findings, we offered insights to encourage and inform future research and intervention that support and engage caregivers to facilitate the AYA’s transition to HIV self-management.

## Supporting information

S1 Text Interview guide. (PDF)

S1 Checklist Inclusivity in global research. (DOCX)
