# Supplementary material for: “I can’t fail. And my son can’t fail.”: Caregiver perspectives on supporting youth to self-manage their HIV in Ndola, Zambia
Source: PLOS Glob Public Health. 2025 Nov 20;5(11):e0005416. doi: 10.1371/journal.pgph.0005416 (PMC12633860; doi:10.1371/journal.pgph.0005416)
Supplement: S1 Text — (PDF) [file pgph.0005416.s001.pdf]

Appendix: In-depth Interview Guide for Caregivers

**Study Title:**	Transitioning Adolescents to HIV Self-Management in Ndola, Zambia
**U.S. Principal Investigator:**	Dr. Julie Denison, Assistant Professor, Johns Hopkins Bloomberg School of Public Health, jdenison@jhu.edu
**Zambia Principal Investigator:**	Dr. Jonathan K. Mwansa, Senior Medical Superintendent, Arthur Davison Children's Hospital, jonathankmwansa@gmail.com
**Study Sponsor:**	USAID/Project SOAR/ADCH/Johns Hopkins Bloomberg School of Public Health
**JHU IRB#:**	IRB00007870

APPROVED
26 OCT 2017
ERES CONVERGE
P/BAG 125, LUSAKA

Date of interview (DD/MM/YY): ____________

Caregiver Participant ID: ________ **Youth Participant ID** ________

Caregiver Age: ______ **Caregiver Sex:** __________

Select youth setting: ☐ **Pediatric** ☐ **Adult** ☐ **Transitioned/Transferred**

Interview start time: ______ **Interview end time:** ______

Interviewer Initials: ______

Introduction: Great, now we may start the interview. We are doing this work to help the clinic improve care for young people. It will also help us understand what young people and caregivers think of the peer-mentoring program. I know it may feel uncomfortable to talk about your youth's HIV status and the things you have discussed in your group meetings. Remember though, this information is confidential. You can also refuse to answer any question. Please feel free to tell us anything about your experiences with the program – both good and bad. There are no "right" or "wrong" answers; we are here to learn from you. Let us start with some questions about your relationship with your youth.

Instructions for Interviewer: *Turn on the recorder. Record your name, the caregiver participant ID, date, current time, and interview location (spoken into recorder).*

☐ **Caregiver-youth relationship**

- Tell me about your relationship with your youth.
- How do you feel about your involvement in your youth's life and health?
  - How, if at all, would you like your youth's behavior to change?
  - What do you think caregivers should do to support their youth living with HIV?

- How do you think the clinic should involve caregivers to support their youth living with HIV?

☐ **Overall Program Impressions**

- What were your experiences with the Project YES peer-mentoring program?
- In your words, what was the purpose of Project YES? What were the peer mentors supposed to do?
- What did you like and dislike about the program?
- How do you think the program helped or did not help you and your youth?

☐ **Experiences with Peer Mentors (from Caregiver perspective)**

- Did you attend the initial orientation meeting with your youth, the peer mentor, and the healthcare provider?
  - Can you talk me through how that meeting went?
  - What did you like/not like?
  - What was helpful/not helpful about this initial meeting?
- What has your youth shared with you about being in Project YES?
- Did you have any concerns about having your youth join the program?
  - Were there any other people in your home who had concerns about your youth joining the program?
- How did you feel about your youth's experience with their peer mentor in this program?
  - Do you feel like this experience was positive or negative? What makes you say that?
- Have you seen any changes in your youth since they started the program?
- What other support could you use, as a caregiver?

APPROVED
26 OCT 2017
ERES CONVERGE
P/BAG 125, LUSAKA.

☐ **Experiences with Caregiver support groups**

- What were your experiences with the caregiver group meetings?
- How many did you attend?
  - What made it easy/hard for you to attend?
  - What were your reasons for attending/not attending?
- What made it easy/hard for you to participate in the discussions during these group meetings?
- How did these group sessions help or not help you support your youth?
- What did you like and dislike about the group sessions?
- How did you relate to the other people in the group?
  - How were they similar to or different from you?
- How did you feel discussing things that can be hard to talk about, like sex or alcohol, in the group meetings?

☐ **Youth's HIV self-management**

- How did the program help or not help **you** to support your youth's HIV management?
- What does a "viral load test" mean to you? What does "viral suppression" mean to you? What does "drug resistance" mean to you?
- What are your experiences helping your youth take care of their HIV – for example, taking their medications or picking up medications?
- Tell me about the last time your youth missed taking their HIV medication, what happened?
- Is your youth able to do the things needed to be healthy (such as take daily medicines) or do they need help? Why?
- What makes it easy or hard for youth to manage their HIV, including taking their drugs and attending clinic appointments
    - [Probe for: use of alcohol? Experiences of violence? Sexual behavior? Mental health? Stigma?]
- What do you wish your youth understood better about their HIV?
    - What do you think would help your youth better take care of his or her health?
- What do you wish your youth understood better about you?

☐ **Logistics**

- What are your thoughts about where the group caregiver meetings were held?
    - Did you feel like you could speak freely? What are some reasons why you did/did not feel that you could speak freely?
    - In the future, where would you recommend these meetings take place?
- For this program, because it is part of research, we provide 50 kwacha to cover transport costs after each program visit. What are your thoughts about participating in a program if there is no transport money available?
    - If you could only come if there was transport money available, how much transport money would you need to get to the meeting if it was held here at this clinic?

☐ **Youth and transitioning to adult care (for caregivers of ADCH-enrolled clients only)**

For caregivers of youth who **have not** transitioned from ADCH to NTH:

- What are your thoughts about the idea of your youth moving to NTH?
    - What are some of your concerns about your youth moving to NTH?
    - What are some potential benefits of your youth moving to NTH?
    - How would you feel about your youth moving to NTH if they had a Peer Mentor at NTH who would meet with them on a regular basis during the first few months that they receive care at NTH?

For caregivers of youth who **have** transitioned from ADCH to NTH:

- What did you think when you learned that your youth was moving to NTH?
- How has it been for your youth to transition from ADCH to NTH?

APPROVED
26 OCT 2017
ERES CONVERGE
P/BAG 125, LUSAKA.

- What has helped make this change easy?
- What has made this change difficult?
- How can this transition process be improved?

- How, if at all, has your role in your youth's care changed since they transitioned to NTH?

☐ **Experiences with youth drug change (if applicable)**

- Did your youth have his or her drugs changed in the past year?
  - When did they change drugs?
- What happened when your youth changed drugs? What did you think of it?
- What has helped your youth adjust to the new medication?
- What has made it difficult for your youth to adjust to the new medication?

☐ **Program Recommendations**

- Would you want this program to continue? Why/why not?
- Would you recommend this program to others? Why/why not?
- How did you feel about participating in a research study like this one?
  - Have you participated in other research studies?
- What would make the program better?
- Is there anything else you would like us to know?

APPROVED
26 OCT 2017
ERES CONVERGE
P/BAG 125, LUSAKA.
