# Supplementary material for: “I can’t fail. And my son can’t fail.”: Caregiver perspectives on supporting youth to self-manage their HIV in Ndola, Zambia
Source: PLOS Glob Public Health. 2025 Nov 20;5(11):e0005416. doi: 10.1371/journal.pgph.0005416 (PMC12633860; doi:10.1371/journal.pgph.0005416)
Supplement: S1 Checklist — (DOCX) [file pgph.0005416.s002.docx]

Inclusivity in global research

PLOS’ policy on inclusivity in global research aims to improve transparency in the reporting of research performed outside of researchers’ own country or community and ensures that PLOS publications reporting global research adhere to high standards for research ethics and authorship. Authors of relevant research articles may be asked to complete the questionnaire below, which outlines ethical, cultural, and scientific considerations specific to inclusivity in global research. This questionnaire may be requested when researchers have travelled to a different country to conduct research, if research uses samples collected in another country, research with Indigenous populations or their lands, or if research is on cultural artefacts. Researchers travelling to another country solely to use laboratory equipment will not normally be required to complete the questionnaire. However, the questionnaire can be requested at the journal’s discretion for any submission – if you have been requested to complete this questionnaire by the PLOS journal you submitted to, please do so.

Please complete the questionnaire below and include this as a Supporting Information file with your manuscript. Note that if your paper is accepted for publication, this checklist will be published with your article in the supporting information files. Please ensure that you reference the checklist in the main body of your manuscript. We suggest adding a subsection ‘Inclusivity in global research’ to your Methods section and adding the following sentence: “Additional information regarding the ethical, cultural, and scientific considerations specific to inclusivity in global research is included in the Supporting Information (SX Checklist)”

The questions have been designed to be applicable to a wide range of study types, and there are subsections for both human subjects research and non-human subjects research. If any of the questions are not relevant to your research please mark them as “N/A” as appropriate.

**Ethical considerations, permits and authorship**

*This section is applicable to all research types.*

Provide details as to who granted permissions and/or consent for the study to take place in the Methods section of your manuscript. This should include the names of **all** ethics boards, governmental organizations, community leaders or other bodies that provided approval for the study. If individuals provided approval refer to these people by their role or title but do not list their name(s).

Reported on page number: 6-7

*“The Project YES! study was approved by the Zambian Ministry of Health through the National Health Research Authority as well as the ethical review boards of ERES Converge in Zambia and the Johns Hopkins Bloomberg School of Public Health in the United States.”*

If there were any deviations from the study protocol after approval was obtained please provide details of these changes in the Methods section of your manuscript.
Did this study involve local collaborators that are residents of the country where the research was conducted or members of the community studied? If you do not have any authors from said communities, please provide an explanation for this below.

Yes, among our co-authors, Ms. Chibesa and Drs. Mwansa and Miti are all residents of Ndola, Zambia.

Reported on page number: NA

Everyone listed as an author should meet PLOS’ criteria for authorship and all individuals who meet these criteria should be included in the author byline, rather than the acknowledgements. For further information please see the journal’s Authorship Policy.

**Human subjects research (e.g. health research, medical research, cross-cultural psychology)**

Did you obtain written informed consent from a representative of the local community or region before the research took place? How did you establish who speaks for the community? Details of written informed consent obtained from study participants should be reported separately in the Methods section of your manuscript.

This research was carried out as a partnership between the Johns Hopkins Bloomberg School of Public Health and the Arthur Davison Children’s Hospital (ADCH) in Ndola, Zambia. We received written approval to conduct the research from the Ministry Of Health’s (MOHs) National Health Research Authority. We also had approval from the MOH’s Provincial and District level health offices and the Nurse-in-Charge of each of the participating health facilities. We also had a research advisory board who reviewed study processes and helping interpret study findings. This research advisory board included youth and caregivers from the community who were not participants in the research study.The intervention itself was also implemented and shaped by youth living with HIV who were trained and paid youth peer mentors.

How did members of the local community provide input on the aims of the research investigation, its methodology, and its anticipated outcome(s)?

As noted above we had a research advisory board that met regulary (a requirement of the USAID/Population Council funder) allowing interactions, review of methods and data, and input into the research study.

When engaging with the local community, how did you ensure that the informed consent documents and other materials could be understood by local stakeholders?

Informed consent forms were pre-tested prior to the start of the study. In addition, the consent form was a part of the consent process. After reading the form together (and giving a copy of the form to the participant) the interviewer would ask the potential participant to share their understanding of the research and potential risks and benefits. We also had youth peer mentors share the findings in the study clinics and worked with them to present the data in meaningful ways understood by the local stakeholders.

Will the findings of the research be made available in an understandable format to stakeholders in the community where the study was conducted (e.g. via a presentation, summary report, copies of publications, etc.)? Please provide details of how this will be achieved.

Yes, the Project YES! study team held dissemination meetings to ensure that the findings were shared and made accessible to stakeholders in Ndola, Zambia. Regarding the present analysis, the lead author presented and discussed findings with members of one of the participating health facilities as well as the Project YES! youth peer mentors.

**Non-human subjects research using specimens/ animals collected as part of the study, or those housed in archival collections. Examples include archaeology, paleontology, botany and zoology.**

Did the permission you obtained from a local authority to perform the study include an agreement on access to outputs and benefit sharing? This may include procedures to enable fair distribution of the benefits and resources arising from the research performed. Please include any details of Prior Informed Consent and Benefit Sharing Agreements obtained. These may be required by field-specific regulations, for example the Convention on Biological Diversity (CBD) and the associated Nagoya Protocol.

NA

If the material used in your study was imported, please A) provide the year it was imported and B) indicate whether permits were obtained to import/export the materials used, C) provide details of any permits obtained. If this information is not available, please indicate this.

NA

If you used archival specimens, please state how the material used in your study was acquired by the institute it is held in and provide details of any permits obtained for the original excavations/ sample collection. If this information is not available, please indicate this.

NA

How was the potential cultural significance of the materials collected in your study to local communities considered in your research design? Were Indigenous peoples and/or local researchers and institutions involved with archaeological excavations / collection of specimens? If so, please provide a description of their involvement.

NA

If your manuscript includes photographs of human remains please indicate whether authors obtained permission from descendants or affiliated cultural communities to do so.

NA
